# Burden on Family Caregivers Caring for Patients with Schizophrenia 

**Published:** 2015-09

**Authors:** Farshid Shamsaei, Fatemeh Cheraghi, Saied Bashirian

**Affiliations:** 1 Behavioral Disorders and Substance Abuse Research Center, Hamadan University of Medical sciences, Hamadan, Iran shamsaei68@yahoo.com; 2 Chronic Diseases (home care) Research Center, Hamadan University of Medical sciences, Hamadan, Iran f_cheraghi@yahoo.com; 3 Department of Public Health, Faculty of Health, Hamadan University of Medical Sciences, Hamadan, Iran s_bashirian@yahoo.com

**Keywords:** *Burden*, *Family*, *Caregivers*, *Schizophrenia*

## Abstract

**Objective:** The aim of this study ‎was to determine the prevalence ‎of the burden reported by family ‎caregivers of Patients with ‎schizophrenia.‎

**Methods:** This cross sectional ‎study involved face-to-face ‎interviews with family caregivers ‎of patients with schizophrenia. ‎Using convenience sampling, ‎‎225 caregivers were selected ‎from Farshchian psychiatry ‎Hospital in Hamadan, Iran from ‎July to September 2012. ‎Measures included patients and ‎caregivers’ demographic ‎variables and caregivers’ burden ‎using the Zarit Burden Interview ‎‎(ZBI). Data were analyzed by ‎SPSS-18 with Pearson ‎correlation and t-test.‎

**Results: **Using the ZBI, we found ‎that 7.6% of the caregivers ‎experienced “no to low” burden, ‎‎23.5% “mild to moderate”, 41.8% ‎‎“moderate to severe” and 27.1% ‎‎“severe” burden. The mean ‎average score of the responses ‎to ZBI was 51.73 (SD: ± 18.23). ‎The level of burden experienced ‎was significantly associated with ‎age, gender, and educational ‎level, relation to care recipient, ‎caregiving duration and duration ‎of schizophrenia illness.‎

**Conclusion:** **‎** **‏** ‏Mental health ‎professionals need to develop ‎more innovative programs for ‎families of schizophrenic ‎patients. Furthermore, as a ‎replacement for supporting the ‎families and easing their ‎burdens, it may be more ‎effective to include them in the ‎health care team by assigning ‎specific tasks and providing the ‎required resources to them to ‎perform such tasks. ‎

Schizophrenia is a chronic psychosis in which the patient losses contact with reality. It is a devastating illness, often resulting in a loss of social functioning in affected individuals. The family remains the major source of care for the patient with schizophrenia and has a profound effect on their illness. Having a patient with schizophrenia in a family also affects the roles and interactions within the family. They face lots of burden including care burden, fear and embarrassment about illness signs and symptoms, uncertainty about the course of the disease, lack of social support, and stigma. Burden refers to the negative impact of the individual’s mental illness on the entire family (1, 2).

Living with a schizophrenic relative is stressful. Studies have demonstrated that family caregivers of persons with severe mental illness experience significant stresses and have a high level of burden (3, 4). The perceived burdens among family caregivers of patients with schizophrenia had been studied in various Regions and cultures. In Europe, a Spanish study described several major effects of caring, which included poor health of family members, disruptions to social and leisure activities and domestic routines, and reduction in household income (5).

 In Italy, Magliano et al. (1999) investigated the burden and coping strategies of key relatives of patients with schizophrenia and found that the levels of burden on key relatives did not differ significantly from those on other relatives (6). A study in Switzerland identified that the most important predictor of burden was the relationship between the caregiver and the patients with exacerbating schizophrenia. There were significant changes in the relationship during the acute phase of the illness. Other determinants of burden included threats, nuisances, time, and restricted social and leisure activities (7). In a Swedish study, family burden and participation in care of relatives to both voluntarily and compulsorily admitted patients were investigated. It was found that interventions for establishing a well-functioning network in families where relatives experienced mental health problems are useful (8).

In Thailand, families preferred to take care of their mentally ill relatives at home. Nevertheless, a qualitative study found that families perceived caring as suffering; “suffering” referred to the negative experiences in caregiving, which included physical burdens, emotional distress, economic problems, stigma about mental illness, and knowledge deficit about mental illness and its symptoms (9). Another qualitative study in Iran revealed that six major themes included fears and anxiety for the future, psychosomatic impact, feeling isolated and loneliness, financial impact, change in lifestyle and family functioning, and lack of support and knowledge in experiences of family member caregivers of bipolar disorder patients (10).

The problem of family burden when caring for schizophrenic patients is a common challenge in both developed and developing countries. Different health care and social systems in different countries may influence family’s commitment to care. Family care burdens are echoed and encountered in many parts of the world (4). Iranian families are characterized through their intimate interpersonal relationships and many interactions among family members. Therefore, illnesses of one family member cause a substantial burden for the whole family. In addition, Iranian families report a low level of formal support services compared with the Western countries (11). However, little research has been devoted to the identification and understanding of this phenomenon among Iranians.

The aim of this study was to determine the prevalence of the burden reported by family caregivers of schizophrenic patients.

## Materials and Method

***Study Design and Participants***

This cross- sectional descriptive study was conducted from July to September 2012. Using convenience sampling method, 225 relatives of schizophrenic patients were selected. They were responsible for caring for schizophrenic patients at home. Convenience sampling means that the participants are easy to locate (12). In this study, convenience sampling was the best method for selecting a sample as we did not have access to the patients’ files, address or telephone number. Participants were living either in rural or urban areas of the district of Hamadan in Iran. The patients were attending regular meetings for behavioral disorders and substance abuse in Farshchian Psychiatry Hospital in Hamadan/Iran. Caregivers had to meet the following criteria for inclusion in the study: (i) To be 18 years or older, (ii) To take care of an ill relative with a diagnosis of schizophrenia according to Diagnostic and Statistical Manual of Mental Disorders (DSM-IV-TR), (iii) To take care of a patient who was under medication and under regular follow up in the outpatient department for the past one year, (iv) To be living with the patient, and (v) And be free of any diagnosed psychiatric illness. 

Exclusion criteria included: (i) The patient had a diagnosis other than schizophrenia or a comorbid diagnosis, (ii) the patient was not on medications, (iii) the caregiver was not living with the patient for at least 12 months, (iv) the caregiver had a history of psychiatric disorder before being a caregiver.

Data were collected using face-to-face interviews conducted by a trained study team. The Socio-Demographics Questionnaire and one scale were used to collect data from the individuals. A detailed description of the instruments is provided below. The researchers introduced themselves to participants before the interview and clearly expressed the purpose of the study. To ensure privacy, the interviews were conducted in a room where the investigator and the participant were alone.

***Measures***

Burden symptoms of caregivers were assessed using the Zarit Burden Interview (ZBI), which was developed to assess caregiver burden in relatives of patients with chronic mental illnesses (13). It is a 22-item instrument that includes the factors most frequently mentioned by caregivers as problem areas in providing care for mentally ill patients. These factors include the caregiver’s health, psychological wellbeing, finances, social life, stigma details, and patient- caregiver relationship. The instrument has a possible score of 0-88, depending on the caregiver’s responses. Responses were rated from 0-4, based on the level of distress. The ZBI scoring was converted into categorical responses in this study. Scores ranging from zero to one were regarded as negative, while scores ranging from two to four were regarded as positive. The instrument was used to assess caregiver burden not only in dementia but also in schizophrenia (14).

The Zarit Burden Interview (ZBI), which provides a comprehensive assessment of both objective and subjective burden is one of the most commonly used burden measures and has been validated in many culturally or ethnically different populations. In the study of Seng et al. in Singapore (2010), the Cronbach’s alpha value was 0.93 and the intra-class correlation for the test-retest reliability was 0.89 (15). The Japanese version of ZBI in the study of Arai et al. (1997) had a good test-retest reliability (r = 0.76) and internal consistency (Cronbach's alpha = 0.93) (16). Test-retest reliability of the Brazilian version of ZBI was 0.80 and the Cronbach's coefficient alpha was 0.77 (17). The instrument was validated in Iran among caregivers of patients with mental disorders. The overall Cronbach’s alpha was 94% and the intra-cluster correlation that was obtained through comparing the overall score of the questionnaire in the pre-test and test phase was 97% (18).

***Statistical Analysis***

Data were analyzed using the Statistical Package for Social Studies (SPSS) software version 18. A descriptive analysis using means with standard deviation, frequency counts and percentages was carried out. Pearson correlation coefficients (r) were employed to address the relationship between caregiver burden and study variables. T-test was used to compare males and females on caregiver burden. The level of statistical significance was set at P<0.05.

Before beginning the study, ethical approval was obtained from the Ethics Committee at the Hamadan University of Medical Sciences, and all participants signed the informed consent to participate in the study.

## Results

The study included 225 caregivers, 39 (17.3%) were spouses, 113 (50.2%) were parents, 53 (23.5%) were children and 20 (8.8%) were siblings. The majority of the caregivers (73.7%) were female. Moreover, 70.7% of the participants were married, 17.3% were single, and 12% were divorced/ widowed. Their age ranged from 21 to 71 with a mean of 53.3 years (SD = 18.7), median was 47 years. In addition, 36.8% held primary school degree, 45.3% held high school diploma, and 18.7% held university degrees. The mean duration of schizophrenia was 9.8 (SD = 6.7) and the mean duration of care giving was 5.2 years (SD = 1.4). Socio-demographic characteristics are presented in Table1.Descriptive and bivariate analyses were performed to determine the degree of caregiver burden. It was observed that respondents reported a varied degree of burden with a mean score of 51.73±18.23. The majority of them (41.8%) experienced moderate to severe burden and 7.6% experienced “no to low” burden, 23.5% “mild to moderate”, and 27.1% “severe” burden. Consequently, 43.1% of the caregivers had experienced feelings of burnout.

When each item on the scale was analyzed, higher scores were found for the following questions: “Do you feel that your relative asks for more help than he/she needs?” (46.7%); “Do you feel embarrassed over your relative's behavior?” (38.7%); and “Are you afraid what the future holds for your relative?” (32.3%); do you feel that your relative seems to expect you to take care of him/her as if you were the only one he/she could depend on (28.3%)?”

Most of caregivers were women, and the degree of burden was highest in women caregivers (Table 2).

Factors affecting caregiver burden are demonstrated in Table 3. Age (elderly), relationship to care recipient (daughter and son), years of chronic illness, and caregiving duration were statistically significant factors that influenced caregiver burden (p<0.05).

There were no statistical significant association between socio-demographic characteristics and occupation and marital status.

**Table 1:** Socio-demographic characteristics of caregivers and patients with schizophrenia

**Variables**	**Caregivers (N/%)**	**Patients (N/%)**
**Age (years)** 30> 31-40 41-50 51-60 >61	30 (13.3) 42 (18.7) 56 (24.9) 64 (28.4) 33 (14.7)	46 (20.4) 61 (27.1) 73 (32.4) 29 (12.9) 16 (7.1)
**Gender** Female Male	166 (73.7) 59 (26.3)	104 (46.2) 121 (53.8)
**Education** Illiterate Primary school Secondary school University or higher	37(16.4) 44 (19.5) 102 (45.3) 42 (17.8)	53 (23.6) 99 (44) 62 (27.5) 11 (4.9)
**Marital Status** Married Single Separate Divorce Widowed	159 (70.7) 37 (17.3) 7 (3.1) 9 (4) 11 (4.9)	96 (42.7) 78 (34.7) 22 (9.8) 21 (9.3) 8 (3.5)
**Occupation** Housewife Skilled worker Unskilled worker Unemployed	59 (26.2) 79 (35.1) 69 (30.7) 18 (8)	6 (29.3) 21 (9.3) 60 (26.7) 78 (34.7)

**Table 2:** Comparison of Males and Females on Caregiver Burden (ZBI)

**Variables**	**M**	**SD**	**T**	**P**
Burden			4.54	.013
Females (n=166)	64.26	16.73		
Males (n= 59)	57.25	16.28		

**Table 3:** The Relationship between Caregiver Demographic Variables and Caregiver Burden

**Variable**	**Caregiver Burden**				
	**Little**	**Mild**	**Moderate**	**Severe**	**P.value**
**Age (years)**	34.45 ± 6.74	39.75 ± 7.94	43.37 ± 9.07	46.37 ± 6.93	r=0.267 P=0.000
Gender Female Male	10 (6%) 7 (11.9%)	41 (24.7%) 12 (20.3%)	70 (42.2%) 24 (40.7%)	45 (27.1%) 16 (27.1%)	r=0.298 P=0.000
**Education** Illiterate Primary school Secondary school University or higher	3 (8.1%) 3 (6.8%) 8 (7.8%) 3 (7.1%)	8 (21.6%) 9 (20.5%) 28 (27.5%) 8 (19.04%)	17 (45.9%) 20 (45.5%) 40 (39.2%) 17 (40.5%)	9 (24.3%) 12 (27.3%) 26 (25.5%) 14 (33.3%)	r=0.034 P=0.011
**Marital Status** Married Single Separate Divorce Widowed	11 (6.9%) 4 (10.3%) 0 (0.00%) 1 (11.1%) 1(9.1%)	42 (26.4%) 4 (10.3%) 2 (28.6%) 4 (44.5%) 1(9.1%)	63 (39.6%) 19 (48.7%) 3 (42.9%) 3 (33.3%) 6(54.5%)	43 (27.04%) 12 (30.8%) 2 (28.6%) 1 (11.1%) 3(27.3%)	r=-0.101 P=0.06
**Occupation** Housewife Skilled worker Unskilled worker Unemployed	3 (5.4%) 8 (10.1%) 6 (8.7%) 0 (0.0%)	9 (15.2%) 17 (21.5%) 19 (27.5%) 8 (44. 4%)	30 (50.8%) 32 (40.5%) 25 (36.2%) 7 (38.9%)	17 (28.8%) 22 (27.8%) 19 (25.5%) 3 (16.7%)	r=0.107 P=0.08
**Relation to care** recipient Spouses Parents Daughter and son Brother and sister	3 (7.7%) 11 (9.7%) 2 (3.8%) 1 (5%)	12 (30.8%) 31 (27.4%) 6 (11.3%) 4 (20%)	16 (41.02%) 50 (44.2%) 20(37.7%) 8 (40%)	8 (20.5%) 21 (18.6%) 25(47.2%) 7 (35%)	r=0.118 P=0.000
**Caregiving duration**	4. 6 ± 2.47	4.98 ± 2.15	5.72 ± 2.68	6.42 ± 3.08	r=0.870 P=0.000
**Duration of illness/years**	3.83 ± 1.14	3.89 ± 1.35	5.32 ± 2.47	6.82 ± 2.47	r=0.403 P=0.000

## Discussion

Current medical policy encourages short-term hospital stay and promotes community care for patients with schizophrenia. Family members are the main support system and shoulder the responsibility for patient care in the community. The personal impact of schizophrenia as a chronic disease needs to be emphasized. Previous research on Iranian family caregivers of patients with schizophrenia has been minimal and has primarily focused on how the caregiver’s affect toward the patient is related to the course of schizophrenia. Thus, little is known about Iranian family caregivers’ burden related to their caregiving roles. The purpose of this study was to determine the prevalence of the burden reported by family caregivers of patients with schizophrenia. Another aim of this study was to determine the socio demographic correlates of the burden on caregivers among relatives of patients with schizophrenia.

 Based on the results, 94 out of 225 caregivers had reported moderate burden and 61 out of 225 had reported severe burden. The results of this study revealed that the caregivers of patients with Schizophrenia experienced significant amount of burden just like most of the studies reported in the literature (19-21). McDonell et al. (2003) reported that family members providing care to patients with schizophrenia experienced high rates of burden (22).

The majority of studies on burden of caregivers of patients with schizophrenia conducted so far report significant burden of caregivers with over 90% of families, experiencing moderate to severe burden (23-24).The burden of care givers of patients with schizophrenia was large and multifaceted. Headmost, there were the direct costs of providing care for patients with schizophrenia. The indirect costs encompassed the loss of productivity through impairments, disability as well as some legal problems including violence. The burden was present in areas like finance, routine activities, family leisure and interaction (23). The result of this study was also consistent with these findings. The behaviour of the person with mental disorders requires that the caretaker places their own needs and wishes after those of the client. The burden on families' ranges from emotional reaction to the illness, the stress of coping with disturbed behaviour, the disruption of household routine, the stigma they are confronted with and the restriction of social activities to economic difficulties. Lauber reported that threats, nuisances, time spent with the affected one, restricted social life and leisure activities are also predictors of burden (25). Data on such issues are essential in organizing services for the primary caregivers and mobilizing financial assistance.

The second purpose of this study was to describe the association between level of burden and other variables. The results of analyses demonstrated that educational status, relation to care recipient and age and duration of illness were all factors affecting caregiver burden.

The salience of caregiver education which is one of the most replicated findings in this field of study (26) was only reported in the domain of "disruption of routine family activities." However, contrary to the reported association between the low level of education and caregivers' burden, this study found a significant association between the high level of education and caregiver's burden. It is possible that higher level education was responsible for greater perception of the complexities involved in care giving.

In this study, it was also found that duration of illness could affect burden of caregivers, which is opposing to the findings of previous studies indicating that higher burden was associated with more hours of contact with the patient (27); and it also contradict with Lasebikan & Ayinde that found an inverse relationship (28). 

In this study, 73.7 percent of caregivers were female. Caregivers are more likely to be women in many parts of the world. For example, in the United Kingdom, about 58% of the caregivers are women (29). Asian studies found that about 70% of family caregivers are females (9). The majority of family caregiving is usually provided by parents, spouses, or relatives. Studies found that most family caregivers of adult clients with schizophrenia are their parents, and they are of older age. In Asian studies, it is also found that caregivers' burden score was positively correlated with their age (4). Our investigation clearly revealed that caregiver burden in older caregivers is higher. However, the result of this study differed from the Mexican Americans' study that caregivers with younger age experienced higher level of family burdens (4). The differences could be related to the traditional Asian culture, which regards the older people in a family as the “heads of household” who have the major responsibility to take care of other family members and are responsible for their health condition (30).

This study has implications for practice, administration, education and research. The analysis of burden of family caregivers provide basic data required for making decisions, future research and generation of interventional strategies, all geared to promote holistic caring. Family interventional programs should be planned on the basis of a careful assessment of the burden experienced, coping strategies, interpersonal skills and social resources of each relative. 

## Limitations

It is important to test the relationships between the various dimensions of burden and more objective health outcomes among caregivers such as mortality and the development of illnesses among caregivers. The findings reported here are based on cross-sectional data; an important avenue for future research will be to replicate these studies with causal structures using longitudinal data.

## Conclusion

Although schizophrenia affects individuals directly, it indirectly affects their caregivers. Therefore, healthcare services for schizophrenia should also incorporate aspects of wellbeing of caregivers. The study concluded that providing care for a chronically ill and/or disabled family member is stressful. Several factors have been identified that could promote a more comprehensive understanding of the cultural experiences inherent to caring for schizophrenia.

Taken as a whole, these findings provide support for emphasizing early community interventions through redesigning in-home services that better meet the social needs of schizophrenic patients and provide more efficacious respite to caregivers.

According to our study findings, health systems need to conduct a mapping of psychosocial provisions for both family caregivers and patients to decrease the family burden rates and increase the possibility of smooth transition to the society. The finding of this study could be used to develop a comprehensive plan to manage such cases in the future.
